# Physician's knowledge, attitudes, and practices regarding seasonal influenza, pandemic influenza, and highly pathogenic avian influenza A (H5N1) virus infections of humans in Indonesia

**DOI:** 10.1111/irv.12428

**Published:** 2016-10-27

**Authors:** Amalya Mangiri, A. Danielle Iuliano, Yunita Wahyuningrum, Catharina Y. Praptiningsih, Kathryn E. Lafond, Aaron D. Storms, Gina Samaan, Iwan Ariawan, Nugroho Soeharno, Jennifer M. Kreslake, J. Douglas Storey, Timothy M. Uyeki

**Affiliations:** ^1^ US Centers for Disease Control and PreventionJakartaIndonesia; ^2^ Influenza DivisionCenters for Disease Control and PreventionAtlantaGAUSA; ^3^ Johns Hopkins Center for Communication ProgramsBloomberg School of Public HealthBaltimoreMDUSA; ^4^ Center for Health ResearchUniversitas IndonesiaJakartaIndonesia; ^5^ Epidemic Intelligence ServiceCenters for Disease Control and PreventionAtlantaGAUSA

## Abstract

Indonesia has reported highest number of fatal human cases of highly pathogenic avian influenza (HPAI) A (H5N1) virus infection worldwide since 2005. There are limited data available on seasonal and pandemic influenza in Indonesia. During 2012, we conducted a survey of clinicians in two districts in western Java, Indonesia, to assess knowledge, attitudes, and practices (KAP) of clinical diagnosis, testing, and treatment of patients with seasonal influenza, pandemic influenza, or HPAI H5N1 virus infections. Overall, a very low percentage of physician participants reported ever diagnosing hospitalized patients with seasonal, pandemic, or HPAI H5N1 influenza. Use of influenza testing was low in outpatients and hospitalized patients, and use of antiviral treatment was very low for clinically diagnosed influenza patients. Further research is needed to explore health system barriers for influenza diagnostic testing and availability of antivirals for treatment of influenza in Indonesia.

## 1 Background

Indonesia is considered an endemic country for highly pathogenic avian influenza (HPAI) A (H5N1) virus among poultry and reported the highest number of fatal human cases of HPAI H5N1 virus infection worldwide (199 cases and 84% mortality) during 2005 to 2015.^1^, ^2^ Most human cases of HPAI H5N1 in Indonesia have occurred in the western part of the main island of Java. The majority of HPAI H5N1 human cases have been associated with poultry exposure such as direct or close contact with sick or dead poultry or visiting a live poultry market, although limited, non‐sustained human‐to‐human HPAI H5N1 virus transmission has likely occurred.^1^, ^3^, ^4^, ^5^, ^6^, ^7^, ^8^ Mortality from HPAI H5N1 virus infection in Indonesia has been associated with late presentation to health care and hospitalization, delayed clinical diagnosis of HPAI H5N1, and late initiation of antiviral treatment.^5^, ^9^, ^10^, ^11^

In addition to sporadic human infections with HPAI H5N1 virus in Indonesia, seasonal influenza A and B viruses circulate among people. Limited surveillance data suggest that seasonal influenza viruses circulate year‐round in Indonesia, with increased activity during November to March, the rainy season months.^12^, ^13^, ^14^, ^15^ However, data on seasonal influenza disease burden in Indonesia are limited. In addition, there are no published data on the impact of the 2009 H1N1 pandemic in Indonesia.

To assess clinicians’ familiarity with clinical management of influenza patients in Indonesia, we conducted a survey of outpatient and hospital‐based physicians in two districts in western Java: East Jakarta and Bogor. The objectives of the survey were to determine the knowledge, attitudes, and practices (KAP) of physicians about clinical diagnosis, testing, and treatment of patients with seasonal influenza, pandemic influenza, or HPAI H5N1 virus infections. In both of these districts, human cases of HPAI H5N1 virus infection had been identified as recently as 2009 prior to implementation of the survey. In this report, we summarize the findings of the KAP survey.

## 2 Materials and Methods

We conducted a cross‐sectional survey of practicing clinicians in western Java, Indonesia. The study population was comprised of outpatient‐ and inpatient‐based physicians providing medical care for adult and/or pediatric patients at government or private sector health facilities in Bogor (peri‐urban and rural communities) and East Jakarta (primarily urban) districts. We utilized a list of all registered physicians from the District Health Offices in the two districts and randomly sampled general primary care clinicians and subspecialists from the list for recruitment. A standardized data collection instrument was developed with 119 questions about influenza KAP. The instrument was piloted with clinicians in Depok, West Java, to confirm the wording, flow, and time spent for each respondent. Study personnel were trained to use the instrument. Following verbal informed consent from clinician participants, face‐to‐face interviews were conducted in Bahasa Indonesia, the Indonesian language, during March to June 2012. We collected demographic information and data on participants’ clinical practice setting, knowledge of influenza signs, symptoms, and complications, outpatient and inpatient influenza diagnosis, testing, treatment, and preventive measures for seasonal influenza, pandemic influenza, and HPAI H5N1, including the H5N1 suspect case definition used in Indonesia. The suspect case definition for HPAI H5N1 in Indonesia includes acute lower respiratory tract illness with fever and recent exposure to poultry or suspected human cases within 7 days of illness onset and is based upon the World Health Organization (WHO) suspect H5N1 case definition.^16^, ^17^ Clinicians were asked about a series of clinical signs and symptoms that are commonly associated with influenza to see whether they thought a particular sign or symptom was related to seasonal influenza, pandemic influenza during the 2009 H1N1 pandemic (2009–2010), or HPAI H5N1 virus infection in hospitalized patients or outpatients. Distinguishing between seasonal and pandemic influenza on clinical grounds may be impossible. However, because of the lack of published data on the 2009 H1N1 pandemic in Indonesia, we were interested whether clinicians were aware of the influenza pandemic and if they had ever diagnosed a patient with 2009 H1N1. Survey responses were entered into a database using epidata version 3.1 (EpiData Association, Odense, Denmark) with double entry to confirm responses, and data were analyzed using IBM Statistical Product and Service Solutions (spss) version 20 (IBM Co. Somers, New York). Descriptive statistics were calculated to analyze the results using spss. Human subjects review for the study was conducted and approved by the Ethics Committee of the Faculty of Public Health, Universitas Indonesia, with reliance approval by the U.S. Centers for Disease Control and Prevention Institutional Review Board.

## 3 Results

### 3.1 Demographic characteristics

Of 1 725 registered physicians, 1 130 (66%) physicians were approached in the two districts and of those 554 (49%) were enrolled in the survey (Table 1). The 595 physicians who were not approached to participate did not have a valid address for contact or were no longer practicing physicians. Of the 554 physicians enrolled, the median age was 41 years (interquartile range (IQR): 32–50 years), 42% were male, 43% were from East Jakarta; 88% were general practitioners and 12% were specialists including pediatricians (3%), internists (3%), and obstetricians/gynecologists (2%). Overall, 71% of physicians reported providing outpatient care only, 2% provided inpatient care only, and 27% provided both outpatient and inpatient care.

**Table 1:** Demographic characteristics of surveyed physicians, two districts in Indonesia, March–June 2012

Physician characteristics	Physicians (N=554)n (%)
Age (years)	
Median (IQR)	41 (32–50)
Gender	
Female	323 (58)
Physician specialty	
General physician	489 (88)
Subspecialty	65 (12)
Obstetrician/Gynecologist	13 (2)
Pediatricians	16 (3)
Internal medicine	14 (3)
Pulmonologist	8 (1)
Cardiologist	2 (0.4)
Neurologist	6 (1)
Ear, nose, and throat (ENT)specialist	6 (1)
Type of care provided	
Outpatient	392 (71)
Inpatient (hospitalized)	13 (2)
Outpatient & inpatient	149 (27)

### 3.2 Reported knowledge about clinical features of influenza

Clinical signs and symptoms associated with seasonal influenza in outpatients reported by physicians were generally similar for the two districts. Fever (96%), cough (85%), runny nose (75%), nasal congestion (64%), and sneezing (58%) were reported by physicians to be associated with seasonal influenza in outpatients (Table 2). Physicians reported that shortness of breath (24%) was a symptom for hospitalized seasonal influenza patients more frequently than in outpatients (7%).

**Table 2:** Physician reported knowledge of signs and symptoms of seasonal influenza, pandemic influenza A(H1N1)pdm09, and HPAI H5N1 virus infections

Clinical signs and symptoms^a^	Seasonal influenzan (%)	A(H1N1)pdm09n (%)	HPAI H5N1n (%)
Outpatients			
Fever	533 (96)	478 (86)	503 (91)
Cough	473 (85)	366 (66)	407 (74)
Runny nose	414 (75)	274 (50)	303 (55)
Nasal congestion	356 (64)	235 (42)	253 (46)
Sneezing	322 (58)	201 (36)	210 (38)
Muscle ache	280 (51)	291 (53)	299 (54)
Sore throat	268 (48)	223 (40)	235 (42)
Shortness of breath	40 (7)	108 (20)	216 (39)
Hospitalized patients			
Fever	525 (95)	468 (85)	499 (90)
Cough	445 (80)	354 (64)	400 (72)
Runny nose	374 (68)	265 (48)	281 (51)
Nasal congestion	322 (58)	231 (42)	244 (44)
Sneezing	280 (51)	188 (34)	202 (37)
Muscle ache	270 (49)	288 (52)	296 (53)
Sore throat	268 (48)	221 (40)	238 (43)
Shortness of breath	131 (24)	185 (33)	301 (54)

a All signs and symptoms can occur with seasonal influenza, pandemic influenza, and highly pathogenic avian influenza (HPAI) A(H5N1); higher percentage indicates more knowledgeable responses.

For pandemic influenza [influenza A(H1N1)pdm09], fever, cough, muscle ache, runny nose, and nasal congestion were less frequently reported by physicians as being signs and symptoms in both outpatients and hospitalized patients (Table 2).

In addition, physicians reported that fever (91%), cough (74%), runny nose (55%), muscle aches (54%), and nasal congestion (46%) were signs or symptoms for suspected HPAI H5N1 in outpatients. For suspected HPAI H5N1 hospitalized patients, physicians more frequently reported that shortness of breath (54%) was a sign of suspected HPAI H5N1 in hospitalized patients compared to outpatients (39%) (Table 2).

### 3.3 Knowledge of seasonal influenza high‐risk groups

Physicians correctly identified children aged <5 years old (92%), immunosuppressed persons (64%), and elderly (64%) as high‐risk groups for seasonal influenza‐associated complications. However, 41% correctly identified persons with chronic lung disease and pregnant women, and few participants (17%) identified persons with heart disease as high‐risk groups for influenza‐associated complications. 76% of physicians incorrectly identified children aged >5 years as a high‐risk group for influenza complications. Overall, the findings were similar for participating physicians in the two districts (data not shown).

### 3.4 Knowledge of HPAI H5N1 suspect case definition and risk factors

Among all clinicians in the two districts, 98% reported having heard of the HPAI H5N1 suspect case definition in Indonesia. However, when asked about what potential exposures physicians would ask of suspected HPAI H5N1 patients, many physicians reported that they would not ask about key risk factors for HPAI H5N1 virus infection. While 59% of physicians reported that they would ask whether a patient had handled dead chickens in the 7 days before illness onset, only 13% reported that they would ask about contact with chickens that died suddenly, and only 9% reported that they would ask about visiting a market where live poultry were slaughtered in the 7 days prior to illness onset to assess potential HPAI H5N1 virus exposures. Twenty‐two percent of physicians reported that they would inquire about slaughtering chickens by patients in the prior 7 days to determine HPAI H5N1 virus exposure. In contrast, to assess exposure to HPAI H5N1 virus, more than 50% of physicians reported that they would ask about uncommon risk factors for HPAI H5N1 virus infection, such as exposures to wild birds or wild bird feces near the home in the past 7 days (53%) or whether the suspected case had provided care to a confirmed HPAI H5N1 case patient (54%).

### 3.5 Practices related to influenza diagnosis, testing, and treatment

Among outpatient‐based physicians, over 90% reported ever making a clinical diagnosis of seasonal influenza (not laboratory‐confirmed), but only 2% reported having diagnosed pandemic influenza, and 4% reported having diagnosed a case of suspected HPAI H5N1 (Table 3). Among physicians providing care for hospitalized patients, 28% reported ever making a clinical diagnosis of seasonal influenza, while only 0.2% reported having diagnosed pandemic influenza, and 1% had diagnosed a suspected case of HPAI H5N1.

**Table 3:** Physician reported practices for the diagnosis, treatment, and testing of seasonal influenza, influenza A(H1N1)pdm09, or HPAI H5N1 virus infections in outpatients and hospitalized patients (N=554)

	Suspected influenza disease	Influenza clinically diagnosed	Influenza treatment prescribed			Influenza testing ordered
			Over the counter fever or pain reducing medicine^a^	Antibiotic	Antiviral	
		n (%)	n (%)	n (%)	n (%)	n (%)
Physicians caring for Outpatients (n=541)	Seasonal Influenza	512 (95)	512 (100)	235 (46)	16 (3)	12 (2)
	A(H1N1)pdm09	9 (2)	5 (56)	4 (44)	2 (22)	4 (44)
	HPAI H5N1	21 (4)	–	–	6 (29)	6 (29)
Physicians caring for Hospitalized patients (n=162)	Seasonal Influenza	46 (28)	39 (85)	30 (65)	2 (4)	8 (17)
	A(H1N1)pdm09	1 (0.1)	1 (100)	1 (100)	1 (100)	1 (100)
	HPAI H5N1	7 (4)	–	–	4 (57)	4 (57)

a Such as paracetamol.

Among physicians who had clinically diagnosed seasonal influenza in the outpatient setting, only 2% had ordered influenza testing, whereas among those who had made such a diagnosis in the inpatient setting, 17% had ordered influenza testing. Among all physicians who indicated that they ordered influenza testing, 57% reported ordering testing for hospitalized patients who were suspected HPAI H5N1 cases compared to 29% for outpatients (Table 3). Among physicians providing care for outpatients, the most common reported reason that testing was not ordered for seasonal influenza in outpatients was because testing was thought to be unnecessary, too expensive, or was unavailable.

Medicines for relief of symptoms and antibiotics were the most frequently reported treatment prescribed for influenza patients (Table 3). Very few physicians reported prescribing antiviral treatment for seasonal influenza in outpatients (3%) or hospitalized patients (4%).

### 3.6 Knowledge and practice related to Influenza non‐pharmaceutical preventive measures

When asked about ways to prevent influenza, more than half of physicians reported that they recommended that outpatients they diagnosed with suspected seasonal influenza should use a face mask (57%), cover their nose and mouth when sneezing and coughing (44%), and wash hands frequently (38%), but only 13% reported that they would tell the patient to avoid close contact with non‐ill persons. Forty‐eight percent of physicians reported that they recommended that outpatients diagnosed with suspected HPAI H5N1 should cover their nose and mouth when coughing and sneezing, and wear a face mask, and 57% recommended avoiding close contact with other persons.

For hospitalized patients diagnosed with seasonal influenza, physicians reported recommending that the patient should use a face mask (76%), cover the nose or mouth when sneezing or coughing (67%), and wash their hands frequently (46%) to reduce influenza virus transmission to others (Table 4). However, only 26% physicians reported recommending that the patient should limit interactions and avoid close contact with healthy persons to prevent spread of influenza. Only 6 (1%) physicians reported diagnosing a hospitalized patient with suspected HPAI H5N1, and of those, only 3 (50%) recommended that the patient should wear a face mask and avoid close contact with healthy persons to prevent further transmission (Table 4).

**Table 4:** Physician practices for recommending non‐pharmaceutical preventive measures for patients diagnosed with suspected seasonal influenza, pandemic influenza, and HPAI H5N1 in outpatients and hospitalized patients

Recommended preventive measures	Seasonal Influenza		A(H1N1)pdm09		HPAI H5N1	
	Outpatients(N=512) n (%)	Hospitalized patients (N=46) n (%)	Outpatients (N=9) n (%)	Hospitalized patients (N=1) n (%)	Outpatients (N=21) n (%)	Hospitalized patients (N=6) n (%)
Frequent hand washing by patients	208 (38)	21 (46)	2 (22)	1 (100)	3 (14)	3 (50)
Patient should cover nose and mouth when coughing and sneezing	245 (44)	31 (67)	5 (56)	1 (100)	10 (48)	3 (50)
Patient should use face mask	313 (57)	35 (76)	6 (67)	1 (100)	11 (52)	3 (50)
Patient should limit interaction with persons who are not sick	144 (26)	12 (26)	6 (67)	1 (100)	6 (29)	1 (17)
Patient should avoid close contact with healthy persons	72 (13)	10 (22)	0 (0)	1 (100)	12 (57)	3 (50)

Of the 28 physicians who reported making a diagnosis of suspected HPAI H5N1 (outpatients: n=21, hospitalized patients n=7), 83% reported wearing a surgical mask to help prevent exposure to a patient with suspected HPAI H5N1 virus infection. However, only 50% reported placing the patient with suspected HPAI H5N1 virus infection in an isolation room.

### 3.7 Practice related to influenza vaccination

Only 11% of all physicians reported they had received influenza vaccination in the previous 12 months. Of those that reported receiving influenza vaccination, 82% reported receiving seasonal trivalent influenza vaccine, and 15% could not recall what kind of influenza vaccine they received.

Reasons reported by physicians for not receiving influenza vaccine included that they believed being vaccinated was not important (47%), they were not interested in vaccination (24%), vaccine was unavailable (19%), vaccine was too expensive (9%), and vaccine was not effective (3%).

## 4 Discussion

In this descriptive survey of physicians providing care for outpatients and hospitalized patients in two districts located in western Java, Indonesia, most physicians reported that they were generally familiar with the signs and symptoms associated with influenza. Nearly all physicians reported making a clinical diagnosis of seasonal influenza in outpatients, but most had not diagnosed influenza in hospitalized patients. Very few physicians reported ever diagnosing a patient with pandemic influenza or with suspected HPAI H5N1 in either outpatients or hospitalized patients. A recent one‐year surveillance study reported that 15.5% of hospitalized patients with severe acute respiratory infection (SARI) tested positive for influenza viruses, including 36% during weeks of peak influenza activity in East Jakarta District, Jakarta, Indonesia.^15^ Together with our findings, this suggests that Indonesian clinicians need to be educated further about the potential for influenza to cause severe illness resulting in hospitalization. Additionally, physicians should be educated on the value of surveillance data to better understand when seasonal influenza viruses are circulating and on the burden of seasonal influenza in Indonesia, such as the results of ongoing enhanced influenza surveillance among SARI patients.^15^

Almost all physicians reported that they had heard of the suspect HPAI H5N1 case definition in Indonesia and were familiar with some risk factors, but most physicians seemed unaware of certain established risk factors for human infection with HPAI H5N1 virus, such as contact with poultry that died suddenly or visiting a live poultry market where poultry are slaughtered.^18^ In contrast, more than half of physicians thought that very uncommon risk factors, such as providing care for a suspected HPAI H5N1 patient and recent exposure to wild birds or wild bird feces near the home, were exposures to ask about to patients with suspected HPAI H5N1 virus infection. Despite practicing medicine in districts where human cases of HPAI H5N1 virus infection have been identified, these findings indicate that much more education of physicians is needed about risk factors for HPAI H5N1 virus infection. Educational training of physicians, such as that conducted in Vietnam, and continuing medical education modules, could improve clinician knowledge of HPAI H5N1 risk factors and clinical management of patients with suspected HPAI H5N1 virus infection in Indonesia.^19^ Furthermore, according to Ministry of Health policy, any suspected case of HPAI H5N1 virus infection should be referred as soon as possible to a designated H5N1 referral hospital for immediate antiviral treatment and H5‐specific testing.^16^

Most physicians who participated in the survey were aware that young children, elderly, and immunosuppressed persons are at increased risk for seasonal influenza complications, but a majority were not aware that pregnant women, and persons with chronic lung or cardiac disease comprise high‐risk groups for influenza complications.^20^ These findings indicate that education of Indonesian clinicians is needed about influenza complications and high‐risk groups, especially to facilitate prevention and prompt treatment of influenza.

Most physicians reported never ordering influenza diagnostic testing and more frequently reported treating influenza patients with non‐specific medications for symptomatic relief and antibiotics. Very few physicians reported prescribing antiviral treatment for the patients. This could be due to unawareness of the clinical benefit of antiviral treatment, lack of availability of antiviral medications for influenza or because Indonesian Ministry of Health policy only recommends antiviral treatment for patients who are suspected or confirmed with HPAI H5N1 virus infection, and for healthcare workers who are providing care for HPAI H5N1 patients.^16^, ^21^ Of the very small number of physicians who reported diagnosing a patient with suspected HPAI H5N1 virus infection, most reported not prescribing antiviral treatment for outpatients and slightly more than half reported prescribing antivirals for hospitalized patients. Observational studies suggest that early antiviral treatment has greater clinical benefit such as mortality reduction compared to no treatment or late treatment for hospitalized influenza patients, including those with HPAI H5N1 virus infection.^9^, ^10^, ^22^, ^23^

Few respondents reported using recommended measures to prevent influenza transmission. Only a small number of physicians reported having received seasonal influenza vaccination in the previous year. This finding is not surprising since no national influenza vaccination program exists in Indonesia. Overall, physicians who made a diagnosis of influenza in a hospitalized patient reported recommending more preventive measures than in outpatients. Of the small number of physicians who reported diagnosing a patient with suspected HPAI H5N1 virus infection, only about half reported placing the patient in an isolation room. These findings suggest that Indonesian physicians might benefit from more education about how influenza viruses are transmitted and of measures to prevent and control influenza in healthcare workers and healthcare settings as recommended by the World Health Organization.^24^, ^25^

There were limitations to this KAP survey. The survey was conducted a few years after the 2009 H1N1 pandemic was declared over and clinicians may not have recalled making a diagnosis of pandemic influenza during 2009–2010. Laboratory testing for distinguishing seasonal influenza from 2009 H1N1 was not widely available and may have limited a diagnosis of 2009 H1N1 during the pandemic period of 2009–2010. Overall participation of eligible physicians from the two districts was low (49% of recruited physicians participated in the survey and 32% of 1750 registered physicians). Therefore, the findings may not be representative or generalizable to all physicians practicing in East Jakarta or Bogor districts, or in Indonesia.

## 5 Conclusions

Overall, a very low percentage of physician participants reported ever diagnosing seasonal influenza, pandemic influenza, or HPAI H5N1 in hospitalized patients. Use of influenza testing was low in outpatients and hospitalized patients, and use of antiviral treatment was very low for clinically diagnosed influenza patients. Further research is needed to explore health system barriers for influenza diagnostic testing and availability of antivirals for treatment of influenza in Indonesia.

## Disclaimer

The findings and conclusions in this manuscript are those of the authors and do not necessarily represent the views of the Centers for Disease Control and Prevention.
